# Evaluation of the residual efficacy of indoor residual spraying with bendiocarb (FICAM WP 80) in six health districts in Senegal

**DOI:** 10.1186/s12936-019-2829-4

**Published:** 2019-06-13

**Authors:** Cheikh Lo, Abdoulaye Kane Dia, Ibrahima Dia, El Hadji Amadou Niang, Lassana Konaté, Ousmane Faye

**Affiliations:** 1 Laboratoire d’Ecologie Vectorielle et Parasitaire, Faculté des Sciences et Techniques, Université Cheikh Anta Diop de Dakar, Dakar, Senegal; 2 Unité d’Entomologie Médicale, Institut Pasteur de Dakar, Dakar, Senegal; 3 Aix Marseille Univ, IRD, AP-HM, MEPHI, IHU-Méditerranée Infection, Marseille, France

**Keywords:** Indoor residual spraying, Bendiocarb, Residual efficacy, *Anopheles coluzzii*, Senegal

## Abstract

**Background:**

From 2011 to 2014, an indoor residual spray (IRS) programme for malaria vectors control was implemented in six health districts in Senegal. The main objective of the present study was to evaluate the efficacy of bendiocarb (FICAM WP 80) sprayed on different wall surfaces and its impact on malaria vectors. The entomological monitoring activities were carried out monthly in five treated sentinel villages and one control untreated village in each district.

**Methods:**

The residual efficacy of bendiocarb applied at a dosage of 0.4 g/sq m was monitored for a period up to 9 months post-IRS using WHO cone bioassay method. This assay consisted to expose 2–5 days old unfed susceptible *Anopheles coluzzii* females to sprayed walls for a period of 30 min. The mortality rates after 24 h post-exposure were estimated and compared between the different types of walls sprayed in each sentinel village.

**Results:**

The results showed that the residual efficacy varied between the different sprayed walls, from one sentinel village to another and between the different campaigns. The FICAM had a residual efficacy of 3–6 months post-IRS on mud and cement wall surfaces. In some cases, the observed mortality rates were much higher than those reported elsewhere particularly during the first campaign in all the six districts.

**Conclusions:**

The FICAM was found to be effective with a residual efficacy varying from 3 to 6 months. If the quality of the IRS application is excluded as a possible explanation of the short efficacy duration, the results suggest at least two rounds of treatments in order to cover the rainy season that lasts 5 to 6 months in the area. Such treatments could be carried out before the intensification of the rains in July and August in order to better cover the transmission period that occurs between late August and October in the area.

## Background

Malaria continues to be a major public health problem throughout the world and particularly in sub-Saharan Africa [1]. According to the latest World Health Organization (WHO) estimates there were 219 million cases of malaria in 2017 of which 92% in the African region [2]. The strategies against malaria involve rapid diagnosis and treatment, and stopping disease transmission through vector control, which aims to prevent, interrupt or at least reduce transmission [3]. Malaria vector control is based predominately on the use of residual insecticides through indoor residual spraying (IRS) and insecticide-treated nets (ITNs) [4]. The use and scaling-up of these methods can significantly decrease malaria morbidity and mortality [5]. The recent recorded results have, therefore, contributed to the consideration of malaria elimination as a feasible objective [6]. However, with the development of insecticide resistance, there are serious concerns about the maintenance of the effectiveness of these control measures [7].

In Senegal, malaria is still a public health problem despite the valuable results obtained during the last years. A significant regression was noted between 2009 and 2016 with an average parasite prevalence range of 1–3% [8]. However, this decline hides disparities with an uneven distribution of the disease in the country. This situation has led to the implementation of a more operational stratification, allowing the adaptation of the control tools to the specificity of the different epidemiological strata.

As part of the Millennium Development Goals (MDG), Senegal, like many other African countries, benefits from the President’s Malaria Initiative [9]. Since 2007 this has permitted the National Malaria Control Programme (NMCP) to implement IRS campaigns in several health districts with the main objective to interrupt or reduce malaria transmission in selected sites [10]. During the IRS campaigns, several insecticides approved by WHOPES were used. Since the beginning of the IRS programme in Senegal, different insecticide molecules and formulations were successively used, the ICON WP 10 from 2007 to 2009, the K-Othrine WG 250 from 2010 to 2011 [11]. Five years of use of pyrethroid insecticides in IRS health districts has targeted vector populations to high insecticide pressure, which resulted to the rise of insecticide resistance, as observed in all the treated areas. Therefore, pyrethroid-based formulations were replaced by bendiocarb (FICAM WP 80) from 2011 to 2014, and by the 300 CS formulation of Actellic [11].

As part of the IRS campaigns, the present study was carried out to monitor and evaluate the residual efficacy of the treatments with the FICAM WP 80 from 2011 to 2014 in the surveyed health districts in Senegal.

## Methods

### Study area

**Fig. 1:**
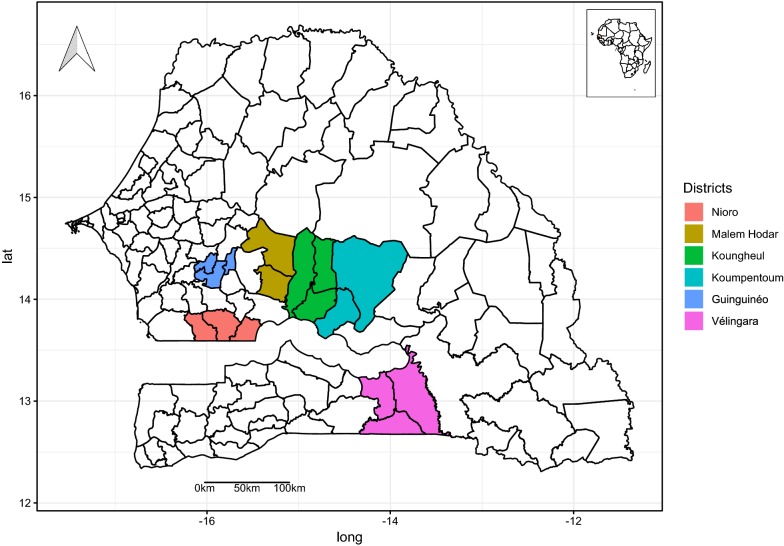
Location of the six health districts

The study was conducted in six health districts in Senegal treated from 2011 to 2014 with the FICAM WP 80 formulation (Bendiocarb). The choice of the districts treated was made in collaboration with the head of each medical health district hospital, considering: (i) the epidemiology of malaria in each sentinel village; (ii) the geographical particularities of the district; (iii) the presence of a health centre where parasitological and clinical studies can be carried out; and, (iv) the existence of previous entomological and/or parasitological data. Table 1 shows the dynamics of different IRS campaigns with the different insecticides used in Senegal since 2007. The health district of Velingara located in the region with the highest malaria annual incidence (> 25‰) [12] belongs to the Sudano-guinean savanna zone. The five other health districts are located in Sudanese area where malaria incidence varies spatially, being moderate (between 5 and 15%) in Nioro and Guinguineo, and as high as in the previous climatic domain (> 25%) in Koumpentoum, Malem Hodar and Koungheul (Fig. 1, Table 2).

**Table 1:** Dynamics of IRS in Senegal from 2007 to 2017

Health districts	Pyrethroids			Carbamates	Organophosphates
	Lambdacyhalothrin		Deltamethrin	Bendiocarb	Pirimiphos-methyl
	ICON WP10	ICON CS 10	K-Othrine WG 250	FICAM WP10	ACTELLIC 300 CS
Vélingara	2007	2008–2009	2010	2011–2013	2014
Guinguinéo	–	–	2010–2011	2011–2012	–
Koumpentoum	–	–	2010	2011–2013	2014–2017
Malem Hodar	–	–	2010	2011–2014	2015–2017
Koungheul	–	–	–	2012–2014	2015–2017
Nioro	2007	2008–2009	2010	2011–2013	2015–2017

**Table 2:** List of the sentinel villages selected in the different health districts treated during the different IRS campaigns in Senegal

Health districts	Sentinel villages	Longitudes (W)	Latitudes (N)	IRS campaigns
Vélingara	Medina Dianghette	12°52′98.5″	13°58′28.9″	2011, 2012, 2013
	Sinthian Koundara	13°15′29.8″	13°54′41.5″	
	Kael Bessel	13°07′76.9″	14°08′0.9″	
	Nemataba	13°13′65.8″	14°04′34.9″	
	Bonkonto	13°1′22.9″	13°55′42.8″	
Nioro	Ndramé Ndimb	15°57′50.2″	13°36′17.7″	2011, 2012
	Thiamène Walo	13°47′2.3″	15°48′4.2″	
	Paoskoto	15°48′4.2″	13°47′2.3″	
	Nguer Français	15°39′3.3″	13°36′14.2″	
	Bamba Diakhatou	16°2′33.1″	14°4′50.5″	
Guinguinéo	Athiou	16°11′28.2″	14°36′18.2″	2011, 2012
	Colobane Lambaye	15°42′31.2″	14°38′44.4″	
	Farabougou	14°15′29.0″	15°56′28.7″	
	Ngathie Naoudé	14°7′51.9″	15°52′55.4″	
	Sakhagne	15°53′8.2″	14°17′39.9″	
Koumpentoum	Altou Fass	13°54′12.9″	14°12′9.1″	2011, 2012, 2013
	Fass Gounas	14°2′43.9″	14°31′13.1″	
	Koumaré	14°22′21.8″	13°54′18.5″	
	Kouthiaba	14°27′17.4″	14°10′38.6″	
	Village 1	14°30′12.9″	13°54′34.5″	
Malem Hodar	Diankhé Souf	15°20′4.71″	14°13′42.8″	2011, 2012, 2013, 2014
	Makka Bella	14°6′36.4″	15°14′3.3″	
	Ndioum Ngainth	14°16′48.2″	14°55′41.9″	
	Niahene	14°1′25.6″	15°11′14.3″	
	Taiba	14°4′57.9″	15°17′57.5″	
Koungheul	Ida Mouride	13°59′17.2″	14°40′54.5″	2012, 2013, 2014
	Keur S. Diébel	13°58′29.7″	14°49′16.6″	
	Touba A. Mbenda	14°7′9.1″	14°45′15.1″	
	Touba Koungheul	14°0′47.5″	14°45′10.9″	
	Pakala	13°49′54.2″	14°56′15.1″	

### Bendiocarb formulation used for IRS treatment

The wet powder formulation of bendiocarb (FICAM WP 80, Bayer), provided by Bayer, was used during IRS campaigns in 2011, 2012, 2013, and 2014, using a treatment dose of 0.4 g/sq m [13]. Bendiocarb is an irreversible inhibitor of acetylcholinesterase and acts on the central nervous system of the insect [14]. It is one of the insecticides recommended by the WHO for use in public health, especially for IRS against malaria vectors [15].

### Bioassays tests

In each sentinel/selected district, the residual efficacy of the treatments was monitored monthly from 2011 to 2014 in six sentinel villages (5 treated and 1 untreated). In each selected village, bioassays were carried on the walls of a total of 5 rooms (4 treated and 1 untreated, chosen as control), randomly selected among different type of human dwellings. Tests were carried out according to the WHO standard cone test procedures for determining the residual efficacy of insecticides on wall surfaces [16] using a susceptible laboratory-reared *Anopheles coluzzii* colony originating from Cameroon. It was maintained for several years, susceptibility for which was checked before each study. Three cones were fixed in three randomly selected walls of the tested rooms, whereas in the control room, one cone was fixed in each of the four walls. Approximately 10 unfed, 2- to 5-day old females were gently introduced into each cone for 30 min. At least 30 and 40 mosquitoes were used per treated and untreated rooms, respectively. In each sentinel village, 120 and 40 mosquitoes were, respectively, exposed to treated and untreated walls for a total of 960 per health district per test round.

After the exposure period, mosquitoes were gently removed from each cone and placed into an individual cardboard cup labelled with corresponding information of each cone per room. The immediate mortality was assessed 20 min post-exposure, then the cups were stored under standard rearing conditions at a temperature of 27 ± 2 °C and a relative humidity of 80 ± 10%; mosquitoes were provided with 10% sugar solution for the 24-h observation period to assess delayed mortality. The travelling time from field to field insectary did not exceed 1 h and caution was taken by holding mosquitoes in a cooler covered with a soaked mop to keep the adequate humidity during the trip.

### Data analysis

The data were recorded in Excel and the analyses performed with R software (version 3.3.1). The residual efficacy of the treatments was evaluated according to WHO criteria procedures for determining the residual effect of insecticides on wall surfaces using 24-h post-exposure mortality rates. Regression curves, showing the evolution of the residual efficacy over the time (months post-spraying), were used for each type of support. Abbot’s formula was used to correct the test groups’ mortality when the mortality rates of the control group were between 5 and 20% [17].

## Results

**Fig. 2:**
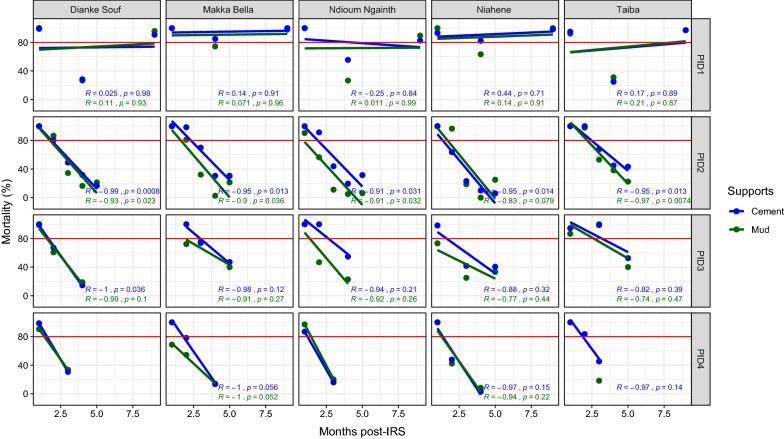
Mortality rate (%) after 24 h of observation on the different wall surfaces treated with Bendiocarb in 2011, 2012, 2013, and 2014 in the district of Malem Hodar. The red horizontal line represents the 80% WHO threshold mortality

The study of the residual efficacy of IRS was carried out in the selected IRS health districts taking into account their specific characteristics. The district of Malem Hodar was treated with FICAM in 2011, 2012, 2013, and 2014. Overall, except for the first IRS campaign, the effectiveness of treatment decreased regularly and rapidly over time with spatial heterogeneity within the district reaching 9 months of residual efficacy in Makka Bella and Niahène during the IRS1 (2011) whatever the type of support. A residual effect was recorded on cement support in both Dianke Souf and Taiba at the beginning and the end of the survey. For the following IRS campaigns, the efficacy of the treatment did not exceed 3 months (Fig. 2).

**Fig. 3:**
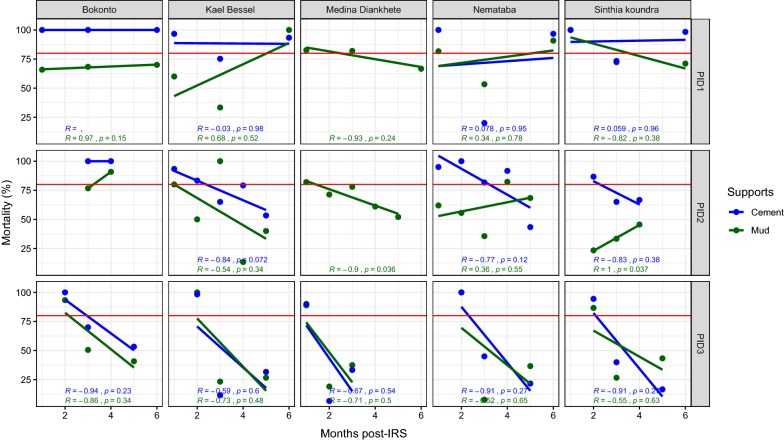
Mortality rate (%) after 24 h of observation on the different wall surfaces treated with Bendiocarb in 2011, 2012 and 2014 in the district of Velingara. The red horizontal line represents the 80% WHO threshold mortality

In the district of Velingara, the Bendiocarb monitored for three successive IRS campaigns (2011, 2012, 2013), revealed that the cement supports were effective up to 6 months in the village of Bokonto. In Kael Bessel, Nemataba and Sinthian Koundara, the efficacy was observed at the beginning and the end of the survey in 2011 (Fig. 3). On mud walls, no efficacy was recorded in Bonkonto. In Kael Bessel and Nemataba, the treatment was effective at the end of the survey whereas in Sinthian Koundara, the effectiveness was observed at the beginning of the survey. During the second year of treatment, the effectiveness was observed only on the cement supports with an effective duration period that did not exceed 2 months (Sinthian Koundara) to 3 months (Bonkonto, Kael Bessel and Nemataba). During the third year of treatment, the effectiveness was observed only during at least 2 months in Bokonto, Kael Bessel, Nemataba and Sinthian Koundara for both the supports on cement and mud (Fig. 3).

**Fig. 4:**
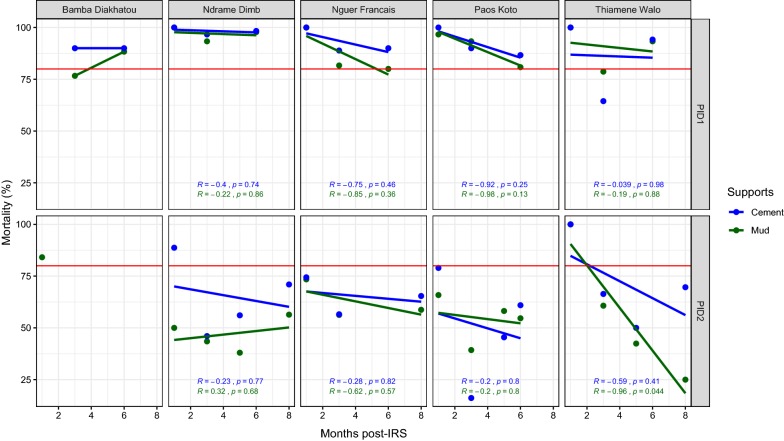
Mortality rate (%) after 24 h of observation on the different wall surfaces treated with Bendiocarb in 2011 and 2012 in the district of Nioro. The red horizontal line represents the 80% WHO threshold mortality

In the district of Nioro, the Bendiocarb was used during two consecutive campaigns (2011 and 2012). In all the villages, the effectiveness of the product remained higher on the cement supports excepted in Thiamène Walo village where it did not exceed 3 months at the beginning of the survey in 2011 (Fig. 4). During the second year of treatment, the product was effective only in Thiamène Walo (both on cement and mud supports), in Ndrame Ndimb (on cement support) and in Bamba Diakhatou (on mud support) at the beginning of the survey. The effectiveness decreased gradually during all post-IRS period in both supports (Fig. 4).

**Fig. 5:**
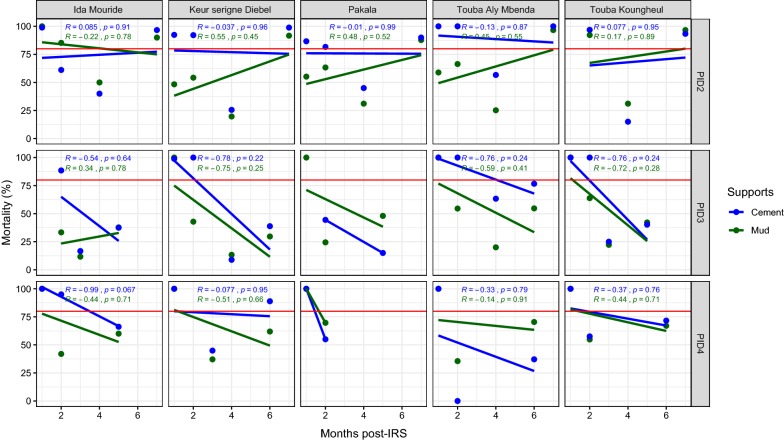
Mortality rate (%) after 24 h of observation on the different wall surfaces treated with Bendiocarb in 2012, 2013 and 2012 in the district of Koungheul. The red horizontal line represents the 80% WHO threshold mortality

From 2012 to 2014, the treatments were done in the district of Koungheul with Bendiocarb. During the first year of treatment, in Touba Aly Mbenda, Keur Serigne Diebel, Touba Koungheul and Pakala, the efficacy was observed up to 2 months on cement support and in Ida Mouride and Touba Koungheul in the mud-plastered supports during 2 months (Fig. 5). During the second year, the effectiveness was observed on cement supports during 2 months in all the villages excepted in Pakala where the treatment in mud support was effective at the beginning of the survey (Fig. 5). The supports on cement retained the residual efficacy of 80% for at least 1 and 2 months in Ida Mouride village during the third campaign, respectively, on mud and cement walls. For the other villages, the effectiveness was observed both on mud and cement walls one month after the treatments (Fig. 5).

**Fig. 6:**
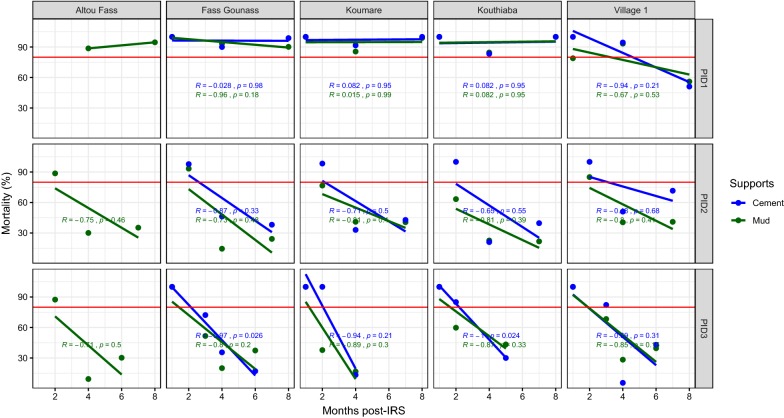
Mortality rate (%) after 24 h of observation on the different wall surfaces treated with Bendiocarb in 2011, 2012 and 2013 in the district of Koumpentoum. The red horizontal line represents the 80% WHO threshold mortality

During the first year of treatment, the cement and mud-plastered supports showed 9 months of effectiveness excepted in Village 1 in the district of Koumpentoum where the residual efficacy was observed up to 4 months on cement and mud supports (Fig. 6). During the second and third years of treatment, the efficacy in mud walls was 2 months in Altou Fass. In Koumare, Kouthiaba, Fass Gounass and Village 1, an efficacy up to 2 months in cement walls was reported in 2012 (Fig. 6). In the third year of treatment, the residual efficacy was observed up to 3 months on cement supports in Village 1 (Fig. 6).

**Fig. 7:**
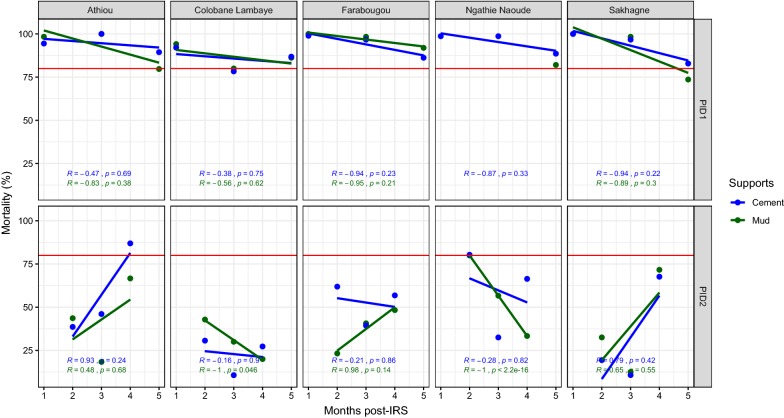
Mortality rate (%) after 24 h of observation on the different wall surfaces treated with Bendiocarb in 2011 and 2012 in the district of Guinguineo. The red horizontal line represents the 80% WHO threshold mortality

In the district of Guinguineo, the first IRS campaign in 2011 showed a residual efficacy of the treatments with bendiocarb in cement and mud walls up to 9 to 10 months (Fig. 7). In 2012, no efficacy was observed excepted in Athiou (on cement support) and Ngathie Naoude (both on cement and mud supports) for, respectively, the eighth and second month after the treatments (Fig. 7).

## Discussion

The residual efficacy of the IRS with the Bendiocarb WP 80 formulation was assessed on different supports (mud and cement) in field conditions in six health districts in Senegal: Velingara and Koumpentoum (2011–2013), Malem Hodar (2011–2014), Guinguineo and Nioro (2011–2012), and Koungheul (2012–2014). During the first campaign the residual efficacy of IRS with bendiocarb lasted up to 6 months post-treatment in Velingara, Nioro and Koungheul health districts. Similar results have been obtained in Mozambique in 2004 [18]. However, IRS efficacy varied within some health districts as previously reported from Zimbabwe in 1991 [19], lasting for only 2–4 months in Village 1 and Thiamène Walo in 2011, located, respectively, in the health districts of Koumpentoum and Nioro. During the following IRS campaigns, the decay rate and the residual efficacy of IRS varied between wall materials, study sites and between IRS campaigns. At health district level, the insecticide decay rate was short (≤ 3 months) in Malem Hodar in 2012, 2013 and 2014, Velingara in 2013, Koumpentoum in 2012 and 2013, as well as in Koungheul in 2013 and 2014. Similar observations have been reported from The Philippines where the post-exposure mortality rates of *Anopheles flavirostris* varied between 75 and 100% up to 3 months post-treatment [20]. Moreover, the residual efficacy of sprays differed between wall structures whatever the surveyed health districts and years, as previously reported from Tanzania and Benin [21, 22]. The observed difference in the decay rate between cement and mud-made walls may be explained by the highest porosity of the mud compared to cement support. The porosity of mud favoured a rapid insecticide intake and its highest decay rate compared to cement structures. Such difference confirms that the entomological efficacy of IRS may be influenced by the structure support due to different intake and decay rates of the insecticide over the time [23].

A surprising increase and rebound in the mortality rate among the exposed mosquitoes was noticed in some health districts for certain years which could be caused by possible relapse of the insecticide in treated surfaces walls due to yet unknown factors. Further investigations are needed to unravel factors underlying such an observation and the mechanisms of the insecticide release several months post-treatment.

The difference observed within and between health districts and between years may be explained by possible operators biases due to lack of good spray competencies of certain sprayers which cause bad spray quality. Therefore, it is highly recommended to carry out refreshing training before any IRS campaign to ensure good spray quality and compliance with operators, communities and environmental safety.

## Conclusions

The Bendiocarb WP 80 formulation was used to control pyrethroid-resistant vectors populations in six health districts as part of the NMCP insecticide resistance management plan. The study revealed that the residual efficacy of IRS with bendiocarb WP 80 formulation lasted in general up to 3 months and varied between localities, health districts and campaigns. If the quality of the IRS application is excluded as a possible explanation of the short efficacy duration, the results suggest that, to cover the rainy season, that lasts 5 to 6 months in the southern regions of the country, at least two rounds of spray are needed to cover the transmission period that occurs between late August and October. Such treatments should be carried out rather before the intensification of rains in July and August in order to cover the period of transmission.

## Data Availability

Data supporting the conclusions of this article are included within the article. Raw data will be made available upon request to the corresponding author.

## Abbreviations

IRS: indoor residual spraying
ITNs: insecticide treated nets
WP: wet powder
WG: wettable granules
CS: capsule suspension
NMCP: National Malaria Control Programme
MDG: Millennium Development Goals
WHOPES: World Health Organization Pesticide Evaluation Scheme
